# Supplementary material for: Motor engagement relates to accurate perception of phonemes and audiovisual words, but not auditory words
Source: Commun Biol. 2021 Jan 25;4:108. doi: 10.1038/s42003-020-01634-5 (PMC7835217; doi:10.1038/s42003-020-01634-5)
Supplement: Supplementary file 2 — Supplementary Information [file 42003_2020_1634_MOESM2_ESM.pdf]

Supplemental Information

SI Note 1. Stimulus details

For the AudWord and AVWord stimuli we generated 350 unique answer sets consisting of a target word and three foils. One hundred sets were used for the AVWord and 100 for the AudWord, with the remainder used for piloting and baseline trials. Within the two word conditions, the same set of four words was used for both the Easy and Hard difficulty levels, but the target word was rotated such that participants heard a unique word on each trial of the experiment. The target words were English nouns, verbs, and adjectives with a mean length of 5.04 letters and mean number of 1.33 syllables per word. We conducted a series of 4x2 ANOVAs to test for differences in condition (AudWord, AVWord) and word list (target, foil1, foil2, foil3) on the following factors: length, log frequency, number of phonological neighbors (with and without homophones), number of phonemes, and number of syllables [1]. We found no differences across condition or word list on any of the factors (**SI Table 1**). Foils were phonological neighbors of the target word, and could differ from the target at any position within the word (e.g. target = “fate”, foils = “fake”, “mate”, “make”).

Phoneme stimuli were vowel-consonant-vowel tokens and participants had to identify the target consonant. Vowels were kept constant within a trial (e.g. target = /aba/, foils = /aka/, /ama/, /ava/), but could vary across trials (vowels included “a”, “i” “o” and “u”). Any VCV stimuli that sounded like English words were removed, and VCVs with “e” were omitted to avoid confusion between the mapping of sound and spelling (e.g. /i/ sounds like “ee” and /e/ sounds like “eh”). This left 80 unique stimuli for the Phoneme condition. Given the more limited stimulus set, the Easy and Hard conditions contained the same targets but had different foils paired with that target (see **Supplementary Data 1**). Within a difficulty level, there were 80

unique targets (i.e. 20 repeated). Our experiment code ensured that same targets were not presented close together in time within or across difficulty levels.

Environmental sounds consisted of sounds like animal calls and natural sounds (i.e. thunder) that do not reflect "doable actions" [2]. One hundred and sixty sounds were gathered from freely available internet sources. In order to ensure that the environmental sounds were identifiable and were recognized as corresponding to the labels that were assigned to them, each sound was assigned to one of three identical free-response norming tasks in Amazon Mechanical Turk [3]. A total of 288 users participated. Some users completed more than one of the three tasks, such that each sound was evaluated by 100 unique participants. In this task, participants clicked a button to play the sound, and then wrote in what they thought they heard. Only sounds with above 50% agreement were chosen, and the target names were selected based on participant responses. In order to approximate the target-foil relationship of the speech stimuli, the foils for the environmental sounds were chosen based on similarity of pitch and temporal characteristics (e.g. target = "bird chirping", foils = "cash register", "teapot", "wind chimes"). It is worth noting that 50% agreement in freely generated responses still translated to a well-recognized sound. For example, a sound like a horse neighing had a high degree of agreement (95% said "horse"), whereas an ambulance siren only had 51% agreement, with the three most common responses being "police", "ambulance", and "siren". Thus, even at 51% agreement, the sound was recognizable from among four options ("ambulance siren", "trumpet", "whale", "alarm clock"). Based on the responses of the norming procedure, 120 unique sounds were used in the final experiment, meaning that each difficulty level had 60 unique targets and 40 repeats. Repeating targets were not presented close together in time within or across difficulty levels. In order to approximate the target-foil relationship of the speech stimuli, the foils for the environmental

sounds were chosen based on similarity of pitch and temporal characteristics (e.g. target = "bird chirping", foils = "cash register", "teapot", "wind chimes"). Like the AudWord and AVWord stimuli, the answer sets were the same between the Easy and Hard levels, with a different sound serving as the target.

SI Note 2. Stimulus recording and preparation

AVWord, AudWord, and Phoneme stimuli were taken from video recordings of a female native English speaker maintaining a neutral facial expression and tone. The recordings took place in a sound-attenuated booth. All audio files were extracted from the video using Adobe Premiere Pro. The pink noise mask was generated in Audacity using the built-in noise generator (amplitude = 0.8)[4]. Pink noise is commonly used as a mask in perceptual experiments[5,6] because it is similar to the temporal envelope of speech[7]. Finally, all auditory stimuli (including the noise mask) were sampled at 44100Hz and root mean square (RMS) normalized using Adobe Audition.

For the AVWord condition, the extracted, normalized audio files were presented simultaneously with the video of the speaker articulating the word. The remaining conditions (AudWord, Phoneme, EnvSound) were presented with a static frame of the video that was morphed to preserve low-level visual properties[8] (**Figure 2**). The goal of the audiovisual condition was to test contributions of both visual and auditory speech during perception. However, during piloting, participants frequently attained 50% accuracy in the AVWord condition when the auditory volume was below perceivable levels. To ensure that participants would not disproportionally rely on the visual input in the AVWord Hard condition, we blurred the mouth of the speaker to obscure fine movements but not overall motion. Notably, prior

evidence shows that even degraded visual speech activates the motor system [9]. The level of blurring was determined by testing pilot participants on silent versions of the AVWord stimuli and selecting a resolution that prevented participants from reaching 50% accuracy using lip movement alone. This blur was applied to all AVWord stimuli, and we measured each participant's lipreading ability in a separate behavioral task. The lipreading task was identical to AVWord trials of the experiment, with the exception that there was no auditory input and the stimuli were not used in the main task.

SI Note 3. Adaptive staircase procedures

Prior to the start of EEG recording, participants first completed a baseline period (20 trials per stimulus type/difficulty combination) using a 1 up 6 down staircase for the Easy condition, and a 1 up 2 down staircase for the Hard condition to set initial SNRs for each condition. Throughout the remainder of the task, on each even numbered trial, the program evaluated the average accuracy of the last 5 trials relative to the desired accuracy threshold for that condition (80% or 50% correct). Stimulus volume was then either adjusted by 0.5 decibels or held constant depending on whether accuracy was above, below, or equal to the desired level. Note that each stimulus type/difficulty combination was adjusted separately, such that if performance fell below 80% on the AudWord Easy trials, the SNR was adjusted for those trials only.

SI Note 4. EEG preprocessing

EEG preprocessing and analysis was conducted in EEGLAB[10]. Raw data were first low-pass filtered (FIR, Hamming window, filter order 330, cutoff frequency 57.5Hz), then down-

sampled to 250Hz, and finally high-pass filtered (FIR, Hamming window, filter order 660, cutoff frequency 2Hz). Bad channels were manually identified and rejected. We next applied two complementary methods of data cleaning and rejection: Artifact Subspace Reconstruction (ASR)[11,12] and independent components analysis (ICA) (for in-depth descriptions of these processes, please refer to Supplementary Materials S1-S4 of Loo et al., 2019)). ASR was applied using the EEGLAB plugin *clean_rawdata* [14]. Following ASR, the rejected channels were interpolated, the data was re-referenced to the average reference. Adaptive mixture independent component analysis (AMICA) was applied to identify stationary brain and non-brain (i.e. artifactual) source activities [15]. The "pcakeep" option was used to adjust the data rank to reflect the number of interpolated channels in each dataset. To estimate the equivalent current dipoles for the scalp projections of each of the independent components (ICs), and the EEGLAB plugins *dipfit()* [16] and *fitTwoDipoles()*[17] were used. Selection of qualified brain independent components was performed in the following way. All individual subject data was loaded into an EEGLAB STUDY structure, and any ICs with dipolar sources outside the brain or accounting for greater than 15% of the residual variance were rejected. Remaining ICs were regarded as qualified brain components and clustered across the group using power spectral density (PSD) precomputed prior to this clustering. Each IC cluster and their underlying individual scalp maps was manually examined, and the those clusters that showed artifactual PSD patterns were marked and rejected. Any subject files with less than 8 ICs remaining were excluded from further analysis, which resulted in the exclusion of two subjects. The ICs belonging to the remaining IC clusters were back-projected into individual channel space. The continuous EEG data were epoched from -2 to 1.5 seconds relative to the onset of the stimulus sound, and subjected to a final round of quality

control and trial rejection procedures, with the final average number of trials per subject per condition shown in **SI Table 2** below.

SI Note 5. Component clustering

The number of component clusters was determined by manually searching the parameter space while evaluating the trade-off between spatial resolution and unique number of participants per IC cluster so that it maximizes the number of unique datasets per cluster while adequately capturing the spatial spread of the dipoles (final number of clusters = 16). The time-frequency data was not used in the clustering procedure to avoid “double dipping” [18]. Dipole locations of all clusters were visualized using the EEGLAB plugin *std_dipoleDensity* with a Gaussian smoothing kernel set to FWHM=15mm.

SI Figures

SI Fig. 1. Average volume per condition

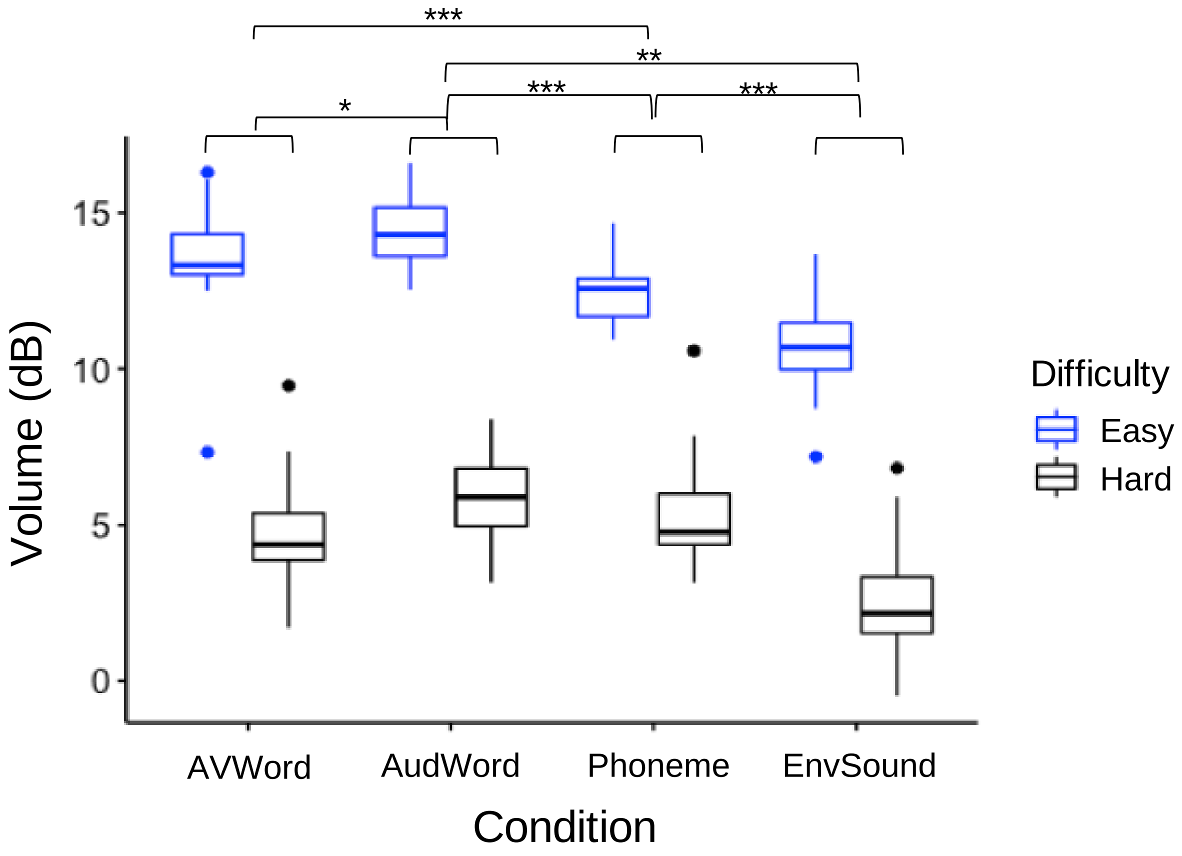

A 4x2 ANOVA with the significant main effects of condition (F(3,187) = 46.12, p < .001) and difficulty (F(1,187) = 1376.78, p < .001). Post hoc comparisons using paired pairwise t-tests (Bonferroni corrected) revealed significant differences in volume between the AVWords and AudWords (p = .006), AVWords and Phonemes (p < .001), AudWords and Phoneme (p < .001), AudWords and EnvSound (p < .001), and Phoneme and EnvSound (p < .001). (Significance levels: *= p<.05, ** = p<.01, *** = p <.001).

SI Fig 2. Overall Task Structure

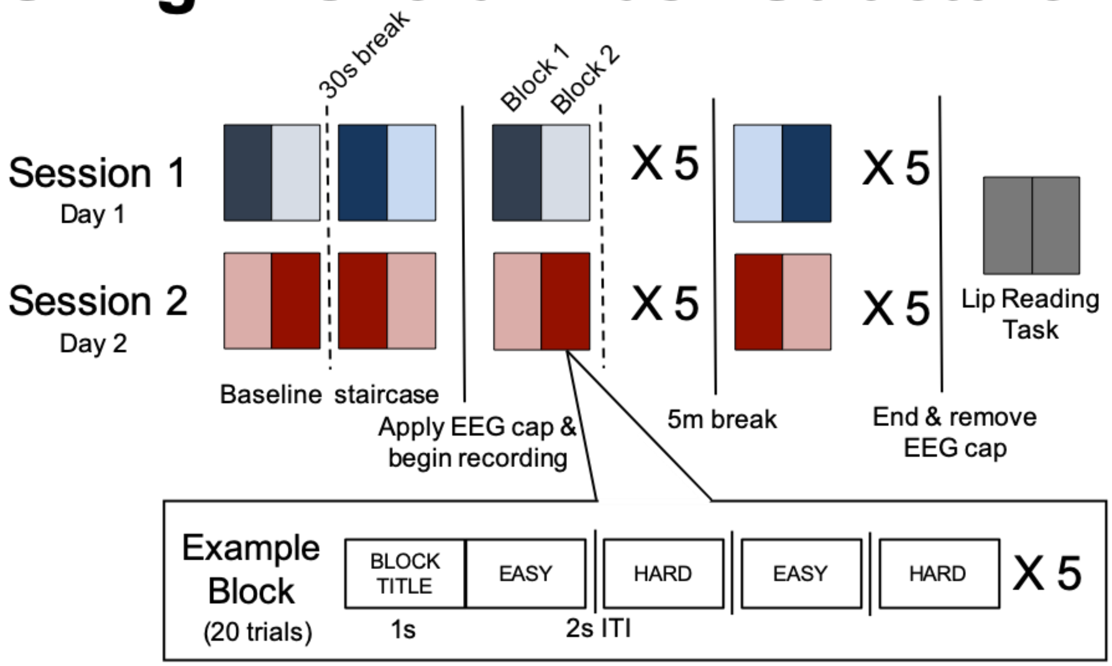

Each session consists of two conditions (either (auditory & audiovisual) or (phoneme & environmental)), and the order of the condition groups is counter balanced. Within a session, condition order is randomized. The lip reading task occurs at the end of the session containing the auditory and audiovisual conditions. Within an individual block, the easy and hard trials alternate. Not including the baseline, there is a total of 800 trials in the entire experiment (100 per condition).

SI Tables

SI Table 1. Results of the 4x2 ANOVAs comparing the lexical stimuli. All values were taken from the English Lexicon Project [1].

	**Length**	**Log Frequency** (HAL)	**Phonological Neighbors** (without homophones)	**Phonological Neighbors** (with homophones)	**Number of Phonemes**	**Number of Syllables**
**Condition** (AudWord, AVWord)	F=2.371, p=.124	F=.324, p=.569	F=.055, p=.815	F=.010, p=.919	F=.035, p=.852	F=.088, p=.767
**Word List** (Target, F1, F2, F3)	F=.515, p=.672	F=.487, p=.691	F=.503, p=.681	F=.309, p=.819	F=.195, p=.900	F=.037, p=.990

SI Table 2. Average number of trials per subject for each condition and difficulty level. Due to the small number of Incorrect Easy trials, analysis of the effect of accuracy collapsed across difficulty, while analysis of the effect of difficulty included correct trials only.

**Stimulus Type**	**Average Number of Trials per Subject**			
	Easy		Hard	
	Correct	Incorrect	Correct	Incorrect
AVWord	78	17	48	48
AudWord	75	19	47	47
Phoneme	71	21	45	47
EnvSound	74	19	49	44

SI Table 3. Mean EEG power (10*log10(μV^2/Hz)) and standard deviation of the independent components for each condition in the left sensorimotor cluster. Values that were significantly different from baseline (results of one-sample t-test) are in bold.

Left Sensorimotor Cluster				
**Accuracy**		**Mean**	**Std. Dev**	**p value**
AVWord	Incorrect	.100	1.195	.406
	Correct	-.198	.940	.090
AudWord	**Incorrect**	**-.233**	**1.011**	**.039**
	Correct	.103	.912	.238
Phoneme	Incorrect	-.029	.920	.684
	**Correct**	**-.200**	**.854**	**.019**
EnvSound	Incorrect	-.076	1.086	.370
	Correct	-.049	.811	.448
**Difficulty**				
AVWord	**Easy**	**-.241**	**.810**	**.025**
	Hard	-.155	1.059	.589
AudWord	Easy	-.027	.945	.820
	**Hard**	**.238**	**.863**	**.037**
Phoneme	Easy	-.142	.785	.263
	**Hard**	**-.257**	**.920**	**.013**
EnvSound	Easy	-.079	.722	.322
	Hard	-.017	.898	.870

SI Table 4. Average volume in dB per condition.

	Average Volume (dB)		
**Stimulus Type**	Easy (SD)	Hard (SD)	Total (SD)
AVWord	13.56 (1.18)	4.71 (1.65)	9.13 (4.79)
AudWord	14.25 (1.09)	5.94 (1.51)	10.09 (4.40)
Phoneme	10.65 (1.38)	2.53 (1.69)	6.59 (4.38)
EnvSound	12.52 (1.06)	5.32 (1.64)	8.92 (3.89)

SI Table 5. Number of independent components (total, for all subjects) per condition in the left sensorimotor cluster.

**Stimulus Type**	**Number of Independent Components per Condition**			
	Stimulus Type x Accuracy		Stimulus Type x Difficulty*	
	Correct	Incorrect	Easy	Hard
AVWord	120	122	60	60
AudWord	122	124	62	60
Phoneme	166	164	83	83
EnvSound	161	164	82	79

*Note, this model contains correct trials only

SI Table 6. Average number of independent components per subject per condition in the left sensorimotor cluster. Standard deviation shown in parentheses. The AVWord and AudWord conditions were tested on one day and Phoneme and EnvSound were tested on the other day, which accounts for numbers of ICs being more similar for conditions tested on the same day.

**Stimulus Type**	**Average Number of Independent Components per Subject**			
	Correct	Incorrect	Easy (correct trials only)	Hard (correct trials only)
AVWord	5.04 (3.75)	5.08 (3.92)	2.5 (1.79)	2.5 (2.02)
AudWord	5.08 (3.86)	5.17 (3.86)	2.58 (2)	2.5 (1.89)
Phoneme	6.92 (4.73)	6.83 (4.80)	3.46 (2.36)	3.46 (2.43)
EnvSound	6.71 (4.81)	6.83 (4.77)	3.42 (2.43)	3.29 (2.40)

SI References

1. Balota, D. A. *et al.* The English Lexicon Project. *Behav. Res. Methods* **39**, 445–459 (2007).
2. Rauschecker, J. P. & Scott, S. K. Maps and streams in the auditory cortex: Nonhuman primates illuminate human speech processing. *Nature Neuroscience* **12**, 718–724 (2009).
3. Buhrmester, M., Kwang, T. & Gosling, S. D. Amazon's Mechanical Turk. *Perspect. Psychol. Sci.* **6**, 3–5 (2011).
4. Audacity v2.3.0. Available at: http://audacityteam.org/.
5. Alho, J. *et al.* Enhanced neural synchrony between left auditory and premotor cortex is associated with successful phonetic categorization. *Front. Psychol.* **5**, 1–10 (2014).
6. Jaekl, P., Pesquita, A., Alsius, A., Munhall, K. & Soto-Faraco, S. The contribution of dynamic visual cues to audiovisual speech perception. *Neuropsychologia* **75**, 402–410 (2015).
7. Yang, M., De Coensel, B. & Kang, J. Presence of 1/ f noise in the temporal structure of psychoacoustic parameters of natural and urban sounds. *J. Acoust. Soc. Am.* **138**, 916–927 (2015).
8. Stojanoski, B. & Cusack, R. Time to wave good-bye to phase scrambling: Creating controlled scrambled images using diffeomorphic transformations. *J. Vis.* **14**, 6–6 (2014).
9. Fridriksson, J. *et al.* Motor speech perception modulates the cortical language areas. *Neuroimage* **41**, 605–613 (2008).
10. Delorme, A. & Makeig, S. EEGLAB: An open source toolbox for analysis of single-trial EEG dynamics including independent component analysis. *J. Neurosci. Methods* **134**, 9–21 (2004).

11. Plechawska-Wojcik, M., Kaczorowska, M. & Zapala, D. The artifact subspace reconstruction (ASR) for EEG signal correction. A comparative study. in *Advances in Intelligent Systems and Computing* **853**, 125–135 (Springer Verlag, 2019).

12. Chang, C. Y., Hsu, S. H., Pion-Tonachini, L. & Jung, T. P. Evaluation of Artifact Subspace Reconstruction for Automatic EEG Artifact Removal. in *Proceedings of the Annual International Conference of the IEEE Engineering in Medicine and Biology Society, EMBS* **2018-July**, 1242–1245 (Institute of Electrical and Electronics Engineers Inc., 2018).

13. Loo, S. K. *et al.* Neural activation and connectivity during cued eye blinks in Chronic Tic Disorders. *NeuroImage Clin.* 101956 (2019). doi:10.1016/j.nicl.2019.101956

14. Miyakoshi, M. clean_rawdata() function. *EEGLAB, Swartz Center for Computational Neuroscience, USCD* (2013). Available at: Makoto Miyakoshi, Swartz Center for Computational Neuroscience (SCCN), Institute for Neural Computation, UC San Diego. (Accessed: 2nd February 2018)

15. Hsu, S.-H. *et al.* Modeling brain dynamic state changes with adaptive mixture independent component analysis. *Neuroimage* **183**, 47–61 (2018).

16. Oostenveld, R., Fries, P., Maris, E. & Schoffelen, J. M. FieldTrip: Open source software for advanced analysis of MEG, EEG, and invasive electrophysiological data. *Comput. Intell. Neurosci.* **2011**, (2011).

17. Piazza, C. *et al.* An automated function for identifying EEG independent components representing bilateral source activity. in *XIV Mediterranean Conference on Medical and Biological Engineering and Computing: MEDICON 2016* (eds. Kyriacou, E., Christofides, S. & Pattichis, C. S.) 105–109 (2016).

18. Kriegeskorte, N., Simmons, W. K., Bellgowan, P. S. & Baker, C. I. Circular analysis in systems neuroscience: The dangers of double dipping. *Nat. Neurosci.* **12**, 535–540 (2009).
