# Supplementary material for: Motor engagement relates to accurate perception of phonemes and audiovisual words, but not auditory words
Source: Commun Biol. 2021 Jan 25;4:108. doi: 10.1038/s42003-020-01634-5 (PMC7835217; doi:10.1038/s42003-020-01634-5)
Supplement: Supplementary file 3 — Description of Supplementary Files [file 42003_2020_1634_MOESM3_ESM.pdf]

Description of Additional Supplementary Files

File name: Supplementary Data 1

Description: Stimulus Appendix. Contains a list of the words, phonemes, and environmental sounds used in each condition of the experiment.

File name: Supplementary Data 2

Description: Behavioral Scores. Contains source data for Figure 3.

File name: Supplementary Data 3

Description: Left Sensorimotor EEG Means. Contains source data for Figure 7.

File name: Supplementary Data 4

Description: Volume by Condition. Contains source data for Supplementary Figure 1.
