# Supplementary material for: Motor engagement relates to accurate perception of phonemes and audiovisual words, but not auditory words
Source: Commun Biol. 2021 Jan 25;4:108. doi: 10.1038/s42003-020-01634-5 (PMC7835217; doi:10.1038/s42003-020-01634-5)
Supplement: Supplementary file 8 — Reporting Summary [file 42003_2020_1634_MOESM8_ESM.pdf]

nature research

Corresponding author(s): Kelly Michaelis, Peter Turkeltaub

Last updated by author(s): Nov 18, 2020

Reporting Summary

Nature Research wishes to improve the reproducibility of the work that we publish. This form provides structure for consistency and transparency in reporting. For further information on Nature Research policies, see our Editorial Policies and the Editorial Policy Checklist.

Statistics

For all statistical analyses, confirm that the following items are present in the figure legend, table legend, main text, or Methods section.

n/a	Confirmed	
☐	☒	The exact sample size (*n*) for each experimental group/condition, given as a discrete number and unit of measurement
☐	☒	A statement on whether measurements were taken from distinct samples or whether the same sample was measured repeatedly
☐	☒	The statistical test(s) used AND whether they are one- or two-sided<br>*Only common tests should be described solely by name; describe more complex techniques in the Methods section.*
☐	☒	A description of all covariates tested
☐	☒	A description of any assumptions or corrections, such as tests of normality and adjustment for multiple comparisons
☐	☒	A full description of the statistical parameters including central tendency (e.g. means) or other basic estimates (e.g. regression coefficient) AND variation (e.g. standard deviation) or associated estimates of uncertainty (e.g. confidence intervals)
☐	☒	For null hypothesis testing, the test statistic (e.g. F, t, r) with confidence intervals, effect sizes, degrees of freedom and P value noted<br>*Give P values as exact values whenever suitable.*
☒	☐	For Bayesian analysis, information on the choice of priors and Markov chain Monte Carlo settings
☒	☐	For hierarchical and complex designs, identification of the appropriate level for tests and full reporting of outcomes
☒	☐	Estimates of effect sizes (e.g. Cohen's d, Pearson's r), indicating how they were calculated

Our web collection on statistics for biologists contains articles on many of the points above.

Software and code

Policy information about availability of computer code

Data collection	Our behavioral task was based on custom code in PsychoPy software (https://www.psychopy.org/). We are happy to share the code for the task upon request.
Data analysis	Data analysis was completed using EEGLAB, a freely-available EEG analysis software package (https://sccn.ucsd.edu/eeglab/index.php). Statistical analysis were completed using R software, also freely available (https://www.r-project.org/).

For manuscripts utilizing custom algorithms or software that are central to the research but not yet described in published literature, software must be made available to editors and reviewers. We strongly encourage code deposition in a community repository (e.g. GitHub). See the Nature Research guidelines for submitting code & software for further information.

Data

Policy information about availability of data

All manuscripts must include a data availability statement. This statement should provide the following information, where applicable:

- Accession codes, unique identifiers, or web links for publicly available datasets
- A list of figures that have associated raw data
- A description of any restrictions on data availability

The data for Figures 3, 7, and SI1 have been provided in Excel format. Additional EEG data supporting the findings of this study is available from the corresponding author upon reasonable request.

Field-specific reporting

Please select the one below that is the best fit for your research. If you are not sure, read the appropriate sections before making your selection.

- [x] Life sciences
- [ ] Behavioural & social sciences
- [ ] Ecological, evolutionary & environmental sciences

For a reference copy of the document with all sections, see nature.com/documents/nr-reporting-summary-flat.pdf

Life sciences study design

All studies must disclose on these points even when the disclosure is negative.

Sample size	Sample size was determined in part by a power analysis conducted in G*Power prior to data collection, which showed that an N of 25 provided 80% power to detect a small to medium effect size (f=.15), as well as through previous studies looking at a similar EEG measure (Jenson, D., Bowers, A. L., Harkrider, A. W., Thornton, D., Cuellar, M., & Saltuklaroglu, T. (2014). Temporal dynamics of sensorimotor integration in speech perception and production: independent component analysis of EEG data. Frontiers in Psychology, 5, 656.; Bowers, A., Saltuklaroglu, T., Harkrider, A., & Cuellar, M. (2013). Suppression of the µ rhythm during speech and non-speech discrimination revealed by independent component analysis: Implications for sensorimotor integration in speech processing. PLoS One, 8(8), e72024.).
Data exclusions	Data from seven subjects was excluded from the final analysis. Of these seven, two were excluded for having fewer than 8 independent components (see SI section 4), and the remaining 5 were excluded for having overall noisy EEG data with large and frequent artifacts that could not be removed by our cleaning procedures.
Replication	Replication was not attempted in this study
Randomization	We used a repeated measures design in which all participants completed all trials of the experiment. The order of blocks and trials within blocks were either randomized or pseudorandomized. Stimulus presentation procedures are further detailed in manuscript section 6.3 and in SI section 1.
Blinding	No blinding was conducted in this study. All participants completed all experimental conditions

Reporting for specific materials, systems and methods

We require information from authors about some types of materials, experimental systems and methods used in many studies. Here, indicate whether each material, system or method listed is relevant to your study. If you are not sure if a list item applies to your research, read the appropriate section before selecting a response.

Materials & experimental systems

n/a	Involved in the study	
x		Antibodies
x		Eukaryotic cell lines
x		Palaeontology and archaeology
x		Animals and other organisms
	x	Human research participants
x		Clinical data
x		Dual use research of concern

Methods

n/a	Involved in the study	
x		ChIP-seq
x		Flow cytometry
x		MRI-based neuroimaging

Human research participants

Policy information about studies involving human research participants

Population characteristics	Data from 24 participants (16 female, 8 male) was included in the final analysis. Mean participant age was 23.9 years, with a range of 21-31 years. Participants were all healthy, right-handed adults with no history of neurologic, psychiatric, or learning disorder, normal hearing, normal or corrected to normal vision. All participants were native English speakers.
Recruitment	Participants were recruited using IRB-approved fliers placed throughout the Georgetown University community as well as the Georgetown Research Volunteer Program, managed by SONA systems. Additional online participants were recruited using Amazon Mechanical Turk.
Ethics oversight	The Georgetown University Institutional Review Board approved the study protocol.

Note that full information on the approval of the study protocol must also be provided in the manuscript.
